# Risk factors for the critical illness in SARS-CoV-2 infection: a multicenter retrospective cohort study

**DOI:** 10.1186/s12931-020-01492-z

**Published:** 2020-10-21

**Authors:** Sijing Cheng, Dingfeng Wu, Jie Li, Yifeng Zou, Yunle Wan, Lihan Shen, Lixin Zhu, Mang Shi, Linlin Hou, Tao Xu, Na Jiao, Yichen Li, Yibo Huang, Zhipeng Tang, Mingwei Xu, Shusong Jiang, Maokun Li, Guangjun Yan, Ping Lan, Ruixin Zhu

**Affiliations:** 1 The Sixth Affiliated Hospital, Sun Yat-sen University, Guangzhou, 510655 China; 2 School of Medicine, Sun Yat-sen University, 510006/Shenzhen, Guangzhou, 518107 China; 3 Putuo People’s Hospital, Department of Bioinformatics, Tongji University, Shanghai, 200092 China; 4 The Third Clinical Medical College of Yangtze University, Jingzhou Hospital of Traditional Chinese Medicine, Jingzhou, 434000 China; 5 Department of Intensive Care Medicine, Dongguan People’s Hospital, Dongguan, 523059 China; 6 Zhongshan School of Medicine, Sun Yat-sen University, Guangzhou, 510080 China; 7 Institute of Digestive Disease, Longhua Hospital, Shanghai University of Traditional Chinese Medicine, Shanghai, 200032 China; 8 Jieyang People’s Hospital, Jieyang, 522000 Guangdong China

**Keywords:** COVID-19, SARS-CoV-2, Intensive care, Ventilator, SOFA score, Age, Dyspnea, Leukocytosis

## Abstract

**Background:**

Prior studies reported that 5 ~ 32% COVID-19 patients were critically ill, a situation that poses great challenge for the management of the patients and ICU resources. We aim to identify independent risk factors to serve as prediction markers for critical illness of SARS-CoV-2 infection.

**Methods:**

Fifty-two critical and 200 non-critical SARS-CoV-2 nucleic acid positive patients hospitalized in 15 hospitals outside Wuhan from January 19 to March 6, 2020 were enrolled in this study. Multivariable logistic regression and LASSO logistic regression were performed to identify independent risk factors for critical illness.

**Results:**

Age older than 60 years, dyspnea, respiratory rate > 24 breaths per min, leukocytosis > 9.5 × 10^9^/L, neutrophilia > 6.3 × 10^9^/L, lymphopenia < 1.1 × 10^9^/L, neutrophil-to-lymphocyte ratio > 3.53, fibrinogen > 4 g/L, d-dimer > 0.55 μg/mL, blood urea nitrogen > 7.1 mM, elevated aspartate transaminase, elevated alanine aminotransferase, total bilirubin > 21 μM, and Sequential Organ Failure Assessment (SOFA) score ≥ 2 were identified as risk factors for critical illness. LASSO logistic regression identified the best combination of risk factors as SOFA score, age, dyspnea, and leukocytosis. The Area Under the Receiver-Operator Curve values for the risk factors in predicting critical illness were 0.921 for SOFA score, 0.776 for age, 0.764 for dyspnea, 0.658 for leukocytosis, and 0.960 for the combination of the four risk factors.

**Conclusions:**

Our findings advocate the use of risk factors SOFA score ≥ 2, age > 60, dyspnea and leukocytosis > 9.5 × 10^9^/L on admission, alone or in combination, to determine the optimal management of the patients and health care resources.

## Background

Prior studies of case series reported that 5 ~ 32% Coronavirus Disease 2019 (COVID-19) patients were critically ill or admitted to intensive care unit (ICU) [1–4]. The varied proportions for ICU admission reflected the severe shortages of ICU beds and ventilators in many countries during the pandemic [5–7], a situation that has led to the withdrawal of ventilators in order to make them available to other patients.

Assessing severity of COVID-19 at the time of admission may allow for optimal management of the patients likely to require critical care and make the best use of the health care resources. A case series study of COVID-19 from a Wuhan hospital with 36 patients admitted to ICU suggested that critically ill patients were older, more likely to have underlying comorbidities, dyspnea and anorexia, but the analysis were not adjusted for confounding factors [1]. Multivariable analysis of the data from two hospitals in Wuhan identified older age, high SOFA (Sequential Organ Failure Assessment) score, and d-dimer greater than 1 μg/ml as risk factors for mortality of adult COVID-19 patients [8].

Aiming for a better understanding of the critical illness in COVID-19, we enrolled 252 patients from 15 hospitals outside Wuhan, and analyzed the clinical features of the critically ill patients, compared to patients with less severe symptoms. We identified higher SOFA score, older age, dyspnea and leukocytosis as the most significant risk factors for poor prognosis of the SAR-CoV-2 (severe acute respiratory syndrome-related coronavirus-2) infection.

## Methods

### Study design

**Fig. 1:**
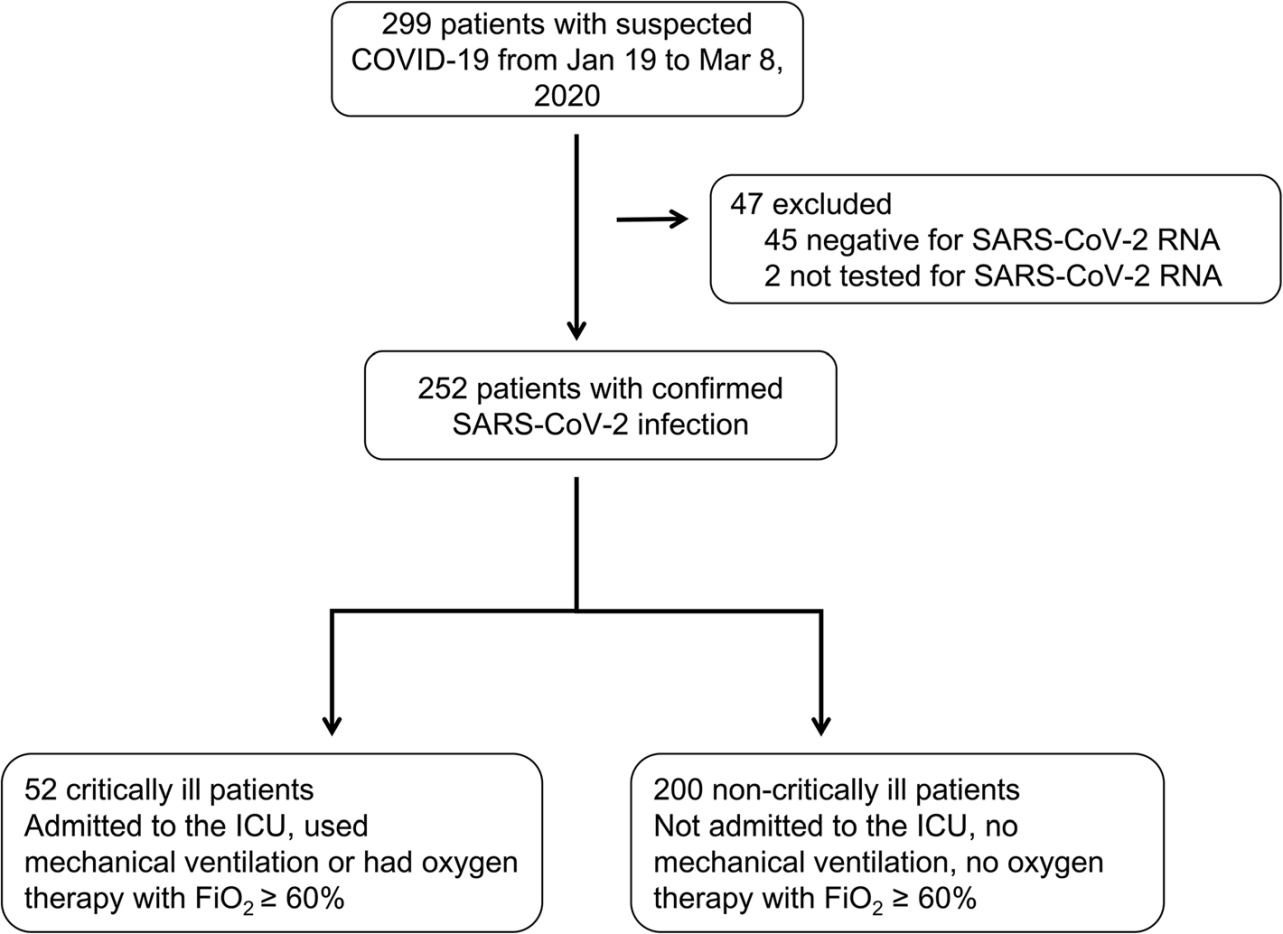
Study flow diagram

We retrospectively studied 252 patients with confirmed COVID-19 admitted to 15 hospitals in Guangdong (2 hospitals), Hubei (3 hospitals), and Jiangxi provinces (10 hospitals), China from January 19 to March 8, 2020 (Fig. 1). The names of the participating hospitals were listed in the footnote to Table 1. Patients were admitted to the hospitals because of fever, cough, dyspnea and chest CT (computed tomography) findings indicating SARS-CoV-2 pneumonia. Diagnosis of COVID-19 was based on positive SARS-CoV-2 real-time PCR test. This study was approved by the Institutional Review Boards of Sun Yat-sen University, and participating hospitals. Informed consent was waived since this study was a retrospective chart review and did not involve any patient tissue or interview.

**Table 1:** Demographics and clinical characteristics of COVID-19 patients on admission

	Total(*n* = 252)	Non-critically ill (*n* = 200)	Critically ill (*n* = 52)	*P* value
Age, years	49(37–61)	45 (35.25–56)	64 (52–73)	**< 0.0001**
< 30	26 (10.30)	25 (12.50)	1 (1.90)	**< 0.0001**
30–39	48 (19.00)	44 (22.00)	4 (7.70)	
40–49	58 (23.00)	54 (27.00)	4 (7.70)	
50–59	50 (19.80)	39 (19.50)	11 (21.20)	
60–69	44 (17.50)	28 (14.00)	16 (30.80)	
70–79	16 (6.30)	5 (2.50)	11 (21.20)	
≥ 80	10 (4.00)	5 (2.50)	5 (9.60)	
Sex				
Female	111 (44.00)	90 (45.00)	21 (40.40)	0.55
Male	141 (56.00)	110 (55.00)	31 (59.60)	
Signs and symptoms				
Fever(≥ 37.3 °C)	179 (71.00)	150 (75.00)	29 (55.80)	**0.006**
Cough	177 (70.20)	136 (68.00)	41 (78.80)	0.128
Myalgia	42 (16.70)	35 (17.50)	7 (13.50)	0.486
Cephalalgia	22 (8.70)	18 (9.00)	4 (7.70)	0.983
Sputum	105 (41.70)	88 (44.00)	17 (32.70)	0.141
Hemoptysis	4 (1.80)	2 (1.20)	2 (3.80)	0.243
Diarrhoea	28 (11.10)	21 (10.50)	7 (13.50)	0.545
Dyspnea	35 (13.90)	6 (3.00)	29 (55.80)	**< 0.0001**
Respiratory rate > 24 breaths per min	26 (10.40)	7 (3.60)	19 (36.50)	**< 0.0001**
Comorbidity				
Hypertension	48 (19.00)	28 (14.00)	20 (38.50)	**< 0.0001**
Diabetes	18 (7.20)	10 (5.00)	8 (15.70)	**0.019**
Digestive tract disease	4 (1.60)	3 (1.50)	1 (1.90)	1
Cardiovascular disease	10 (40)	4 (2.00)	6 (11.50)	**0.006**
Cerebrovascular disease	3 (1.20)	1 (0.50)	2 (3.80)	0.109
Malignancy	4 (1.60)	1 (0.50)	3 (5.80)	**0.029**
Liver disease	6 (2.40)	3 (1.50)	3 (5.80)	0.198

Data are median (IQR) or n (%). p values comparing critically ill and non-critically ill are from Mann-Whitney U test, χ^2^ test or Fisher’s exact test, as appropriate

Medical records of COVID-19 patients were accessed from Jingzhou Hospital of Traditional Chinese Medicine (61 cases), Jianli Hospital of Traditional Chinese Medicine (41 cases), Jingzhou Central Hospital (21 cases), Dongguan People’s Hospital (14 cases), Jieyang People’s Hospital (8 cases), Shangrao People’s Hospital (12 cases), Shangrao No.2 People’s Hospital (3 cases), Poyang People’s Hospital (53 cases), Yugan People’s Hospital (3 cases), Wuyuan People’s Hospital (5 cases), Dexing People’s Hospital (3 cases), Guangfeng People’s Hospital (16 cases), Yushan People’s Hospital (9 cases),Yanshan People’s Hospital (2 cases), Wannian People’s Hospital (1 case)

Demographic data, clinical features, laboratory, and radiological characteristics and treatments and outcomes were obtained from electronic medical records. The data were reviewed by physicians. Laboratory data on admission were collected from the first-time examination upon admission (within 24 h after admission), while the laboratory data before discharge were collected from the last-time examination before discharge or death. Demographic data included age, gender, and co-morbidities including hypertension, diabetes, cardiovascular disease, chronic obstructive pulmonary disease, chronic liver disease and malignancy. Clinical data included vital signs such as temperature, heart rate, blood pressure, respiratory rate and oxygen saturation, fever, cough, dyspnea, sputum production, diarrhea, bloody stool, myalgia and haemoptysis. Laboratory data included complete blood count with differential (white blood cell, lymphocyte, neutrophil, monocyte, and platelet), markers for coagulation function (activated partial thromboplastin time (APTT), fibrinogen, and d-dimer), infection-related biomarkers (Erythrocyte sedimentation rate (ESR), procalcitonin, C-reactive protein (CRP), and neutrophil-to-lymphocyte ratio (NLR)), and other blood biochemistry measurements (lactate dehydrogenase (LDH), creatine kinase (CK), creatinine, blood urea nitrogen (BUN), aspartate transaminase (AST), alanine aminotransferase (ALT), total bilirubin and SOFA score).

Treatment of the infection followed the Diagnosis and Treatment Guideline for COVID-19, National Health Commission of the People’s Republic of China. Patients were routinely given antibiotics, usually Moxifloxacin, and antivirus drugs, usually Lopinavir and Ritonavir.

The hospital course was reviewed for severity of disease. Critically ill patients were defined as those admitted to the ICU requiring mechanical ventilation, or had a fraction of inspired oxygen (FiO_2_) of at least 60% [7, 9]. Indications for mechanical ventilation include aggravating or persistent dyspnea after oxygen supplementation, ARDS, hypercarbic respiratory failure, and hypoxemic respiratory failure. The ventilation mode was synchronized intermittent mandatory ventilation (SIMV). The respiration parameters for these patients were: tidal volume, 400-450 ml; FiO2, 50–63%; respiration frequency, 12–16/min; positive end-expiratory pressure (PEEP), 4-10 cm H2O; inspiratory-to-expiratory ratio, 1:1.5–1:2; inspiratory time, 1.25–1.67 s; and inspiratory flow, 2-5 L/min.

### Statistical analysis

Continuous variables were compared using Mann-Whitney U test. Categorical variables were compared with the chi-square test or the Fisher exact test when appropriate. SPSS (Statistical Package for the Social Sciences) version 24.0 software (SPSS Inc) was used for Mann-Whitney U, chi-square and the Fisher’s exact test. Post-hoc power analyses were performed with G*Power (version 3.1.9.2). Age was transformed to categorical variable according to the scheme described in Table 1. Other continuous variables, such as laboratory data, were transformed to categorical variables based on reference values (Supplementary Table S1). Univariable and multivariable logistic regressions were performed to calculate odds ratio (OR) for critical illness and the 95% confidence intervals (CIs) in R (version 3.6.1). Independent risk factors were identified after adjusting for potential confounders (Supplementary Table S1). LASSO logistic regression was performed to select the optimal risk factors for the prediction of critical illness with “glmnet” packages in R (version 3.6.1). All statistical tests were two sided, with *p* values of < 0.05 considered to be statistically significant.

## Results

### Demographics and clinical characteristics

We enrolled 252 SARS-CoV-2 RNA positive COVID-19 patients admitted between January 19 and March 6. Fifty-two patients (20.6%) were critically ill (Table 1). Compared to non-critically ill patients, the critically ill patients were significantly older, with a median (IQR) age of 64 (52–73), compared to 45 (35.25–56) of non-critically ill patients. Although more of our patients were male, the gender ratio of the critically ill patients was similar to that of non-critically ill. On admission, the critically ill patients more often presented dyspnea (29 patients, 55.8%) and elevated respiratory rate (Respiratory rate > 24 breaths per min, 19 patients, 36.5%), compared to 6 patients (3%) and 7 patients (3.6%), respectively, of the non-critically ill. On the other hand, critically ill patients less frequently presented fever on admission.

Critically ill patients more often had comorbidities than non-critically ill patients (Table 1). Twenty (38.5%) of the critically ill patients had hypertension, compared to 28 patients (14%) of non-critically ill. Similarly, more of the critically ill patients presented diabetes, cardiovascular disease and malignancy, compared to the non-critically ill patients.

### Laboratory and radiographic findings

On admission, markers for coagulation function APTT, fibrinogen and d-dimer were consistently higher in critically ill patients, compared to non-critically ill patients (Table 2). Inflammatory biomarkers ESR, procalcitonin, CRP and NLR were markedly and consistently higher in critically ill patients compared to non-critically ill patients (Table 2). Note that procalcitonin is a marker for bacterial and fungal infection, but not for viral infection. Many of the markers for cell, tissue and organ damage including LDH, BUN, AST, ALT and total bilirubin were higher in the critically ill patients compared to non-critically ill patients (Table 2). Before discharge, laboratory tests were performed to ascertain that patients were SARS-CoV-2 RNA negative and that inflammatory and tissue injury markers were in the normal ranges. It was a surprise that not much difference was observed when we compared the laboratory data collected on admission with those collected before discharge (Supplementary Table S3).

**Table 2:** Laboratory and radiographic findings of COVID-19 patients on admission

	Reference values	Total (*n* = 252)	Non-critically ill (*n* = 200)	Critically ill (*n* = 52)	*p* value
**Laboratory findings**					
White blood cell count (X 10^9^/L)	3.50–9.50	5.05 (4.02–6.83)	4.85 (3.88–6.30)	6.45 (4.53–10.91)	**< 0.0001**
Lymphocyte count (X 10^9^/L)	1.10–3.20	1.03 (0.72–1.41)	1.12 (0.74–1.51)	0.79 (0.58–1.07)	**0.0003**
Neutrophil count (X 10^9^/L)	1.80–6.30	3.28 (2.36–4.75)	3.05 (2.31–4.19)	4.87 (2.86–8.37)	**< 0.0001**
Monocyte count (X 10^9^/L)	0.10–0.60	0.36 (0.23–0.54)	0.36 (0.22–0.55)	0.36 (0.24–0.54)	0.654
Platelet count (X 10^9^/L)	125.00–350.00	176.50 (141.00–217.00)	174.50 (140.75–205.25)	194.00 (148.00–274.50)	0.063
NLR	0.78–3.53	3.10 (2.01–5.62)	2.74 (1.92–4.33)	6.51 (2.54–14.12)	**< 0.0001**
APTT (s)	21.00–37.00	30.20 (25.56–35.50)	29.04 (25.41–34.43)	34.20 (28.61–36.39)	**0.003**
FIB (g/L)	2.00–4.00	3.32 (2.68–4.10)	3.20 (2.60–3.88)	3.70 (3.11–4.86)	**0.0005**
D-dimer (μg/mL)	0.00–0.55	0.40 (0.28–0.61)	0.37 (0.25–0.55)	0.50 (0.31–1.30)	**0.007**
ESR (mm/1 h)	0.00–30.00	24.00 (12.00–40.00)	20.00 (9.50–38.00)	31.00 (23.75–45.25)	**0.002**
PCT (ng/mL)	0.00–0.50	0.11 (0.06–0.26)	0.10 (0.05–0.25)	0.24 (0.10–0.37)	**< 0.0001**
CRP (mg/L)	0.00–10.00	15.75 (5.15–43.82)	13.02(4.75–36.39)	24.70 (6.89–100.19)	**0.001**
LDH (U/L)	91.00–230.00	193.40 (156.75–239.15)	188.00 (151.00–236.00)	226.00 (183.00–323.10)	**0.001**
CK (U/L)	25.00–200.00	99.00 (68.45–175.00)	95.00 (70.00–170.50)	150.00 (47.85–186.50)	0.68
Creatinine (μmol/L)	44.00–112.00	71.90 (59.00–85.93)	71.40 (59.00–85.47)	74.19 (62.05–86.00)	0.829
BUN (mmol/L)	2.50–7.10	4.35 (3.40–5.66)	3.92 (3.25–5.37)	5.41 (4.16–6.90)	**< 0.0001**
AST (U/L)	0.00–40.00	31.00 (24.10–43.75)	31.00 (24.00–41.00)	37.00 (25.70–56.00)	**0.018**
ALT (U/L)	0.00–50.00	31.00 (17.00–45.40)	29.00 (15.00–42.50)	36.45 (22.48–56.03)	**0.01**
TBIL (μmol/L)	3.00–21.00	10.40 (7.60–16.85)	9.80 (7.30–13.80)	17.20 (8.65–23.53)	**< 0.0001**
SOFA score		1.00 (0.00–3.00)	1.00 (0.00–1.25)	4.00 (3.00–5.00)	**< 0.0001**
**Imaging features**					
Ground-glass opacity		62 (24.60)	50 (25.00)	12 (23.10)	0.774
Unilateral pulmonary abnormality		45 (17.90)	40 (20.00)	5 (9.60)	0.082
Bilateral pulmonary abnormality		179 (71.00)	145 (72.50)	34 (65.40)	0.314

Data are median (IQR) or n (%). p values are from Mann-Whitney U test, χ^2^ test, or Fisher’s exact test, as appropriate. *NLR* Neutrophil-to-lymphocyte ratio, *APTT* Activated partial thromboplastin time, *FIB* Fibrinogen, *ESR* Erythrocyte sedimentation rate, *PCT* Procalcitonin, *CRP* C-reactive protein, *LDH* Lactate dehydrogenase, *CK* Creatine kinase, *BUN* Blood urea nitrogen, *AST* Aspartate transaminase, *ALT* Alanine aminotransferase, *TBIL* Total bilirubin, *SOFA* Sequential Organ Failure Assessment

Although differences were observed in laboratory findings on admission between critical and non-critical patients, and all critical patients were SARS-CoV-2 RNA positive and presented respiratory symptoms of various degrees, the majority of laboratory findings on admission for critical patients did not exceed the normal ranges. That was unlikely erroneous observations as similar observations were reported by different participating hospitals. For example, inflammation and tissue injury markers procalcitonin, creatinine and BUN were in the normal ranges for most of the critical patients from different hospitals (Supplementary Table S4) Chest CT results showed that 179 patients (71%) exhibited bilateral pulmonary infiltration, while 62 patients exhibited ground-glass opacity (24.6%) and 45 patients (17.9%) unilateral pulmonary infiltration (Table 2). Although not statistically significant, it was surprising and interesting that more non-critical patients had abnormalitites with CT than critical patients on admission. Again, this is not likely an erroneous observation because of similar data from different participating hospitals. In Supplementary Table S5, we summarized the imaging results from 3 hospitals where both critical and non-critical patients were reported. In all three hospitals, similar percentages of non-critical and critical patients presented abnormalities in CT scans. Our data indicated that critical patients may not present more severe CT results on admission. It is expected that the CT abnormalities are more pronounced and prevalent when severe symptoms developed in critical patients. However, for many critical patients, the only available CT results were collected on admission and before discharge.

### Treatments and outcomes

Except for two critically ill patients, all other patients were given antiviral medicine (Table 3). More critically ill patients (47 patients, 90.4%) were given antibiotics, compared to non-critically ill patients (155 patients, 77.5%). More of the critically ill patients were treated with corticosteroids, immunoglobulin and albumin. In contrast, fewer critical patients were treated with a traditional Chinese medicine, the Lung Cleansing and Detoxifying Decoction, which is an extraction from a mixture of 21 herbal plants. Our data support a protective role of this therapy for COVID-19 [10].

**Table 3:** Treatments and outcomes of COVID-19 patients

	Total (*n* = 252)	Non-critically ill (*n* = 200)	Critically ill (*n* = 52)	*p* value
**Treatments**				
Antiviral treatment	250 (99.20)	200 (100.00)	50 (96.20)	**0.042**
Antibiotics	202 (80.20)	155 (77.50)	47 (90.40)	**0.038**
Corticosteroids	28 (11.10)	15 (7.50)	13 (25.00)	**0.0003**
Intravenous immunoglobin	11 (4.40)	1 (0.50)	10 (19.20)	**< 0.0001**
Albumin	7 (2.80)	0	7 (13.50)	**< 0.0001**
Traditional Chinese medicine	192 (76.20)	160 (80.00)	32 (61.50)	**0.005**
Oxygen therapy	200 (79.40)	158 (79.00)	42 (80.80)	0.779
Mechanical ventilation	10 (4.00)	0	10 (19.20)	**< 0.0001**
ECMO	4 (1.60)	0	4 (7.70)	**0.002**
**Outcomes**				
ARDS	21 (8.40)	1 (0.50)	20 (38.50)	**< 0.0001**
ICU admission	43 (17.10)	0	43 (82.70)	**< 0.0001**
Death	6 (2.40)	0	6 (11.50)	**< 0.0001**
Time from illness onset to dyspnea, days	6 (2.75–9.25)	5.00 (1.50–7.50)	7 (3–10)	0.294
Time from illness onset to ARDS, days	9 (6.50–13.50)	2	9.50 (7–13.75)	0.137
Time from illness onset to ICU admission, days	10 (6–18)	/	10 (6–18)	
length of hospital stay	17 (14–20)	17 (14–19)	21.50 (17–27.75)	**0.001**
Time from hospitalization to death	8.50 (4–12)	/	10 (6–18)	

Data are median (IQR) or n (%). *p* values are from χ^2^ test or Fisher’s exact test, as appropriate. *ECMO* Extracorporeal membrane oxygenation, *ARDS* Acute respiratory distress syndrome, *ICU* Intensive care unit

Similar portions of the critically and non-critically ill patients required supplementary oxygen, although all the patients requiring mechanical ventilation and ECMO (extracorporeal membrane oxygenation) were critically ill.

All 200 non-critical patients survived; while 6 out of the 52 critical patients died. The other 46 critical patients survived. Twenty (38.5%) critical patients developed acute respiratory distress syndrome (ARDS), compared to one non-critical patient who developed ARDS (Table 3). All 43 patients admitted to ICU were critically ill, and 6 of them died. Median time from illness onset to dyspnea, ARDS, ICU admission, and death were 6, 9, 10 and 8.5 days, respectively.

### Risk factors associated with critical illness

Based on the published work on SARS-CoV and SARS-CoV-2 infections, and the differential clinical presentations and outcomes between critically ill and non-critically ill patients in our study, we conducted univariable and multivariable logistic regression analysis to identify the potential risk factors for critical illness in COVID-19.

Higher proportions of older patients were critically ill, with an odds ratio (OR, (95%CI) of 2.03 (1.61–2.62) indicating an over 100% increase in the risk of developing critical illness for every additional 10 years in age (Table 4). Age older than 60 years was identified as a major risk factor for critical illness.

**Table 4:** Risk factors associated with critical illness

	Univariable OR (95%CI)	*p* value	Multivariable OR (95%CI)^a^	*p* value
**Demographics and clinical characteristics**				
Age, years	2.03 (1.61–2.62)	**8.92E-09**		
Baseline(< 30)	1.00 (ref)			
30–39	2.27 (0.31–45.77)	0.473534		
40–49	1.85 (0.26–37.22)	0.590015		
50–59	7.05 (1.25–132.86)	0.069253		
60–69	14.29 (2.62–267.08)	**0.012654**		
70–79	55.00 (8.15–1132.20)	**0.000512**		
> 79	25.00 (3.18–538.98)	**0.007295**		
Female sex (vs male)	1.19 (0.65–2.24)	0.58		
Dyspnea (vs not dyspnea)	40.56 (16.19–117.96)	**1.28E-13**	46.01 (15.36–169.48)	**2.30E-10**
Respiratory rate > 24 breaths per min (vs respiratory rate ≤ 24 breaths per min)	15.63 (6.34–42.77)	**1.07E-08**	5.84 (1.53–23.01)	**0.00994**
**Comorbidity present (vs not present)**				
Hypertension	3.84 (1.92–7.64)	**0.000123**	1.45 (0.64–3.19)	0.365
Diabetes	3.53 (1.28–9.493)	**0.0121**	1.74 (0.58–5.02)	0.307
Digestive tract disease	1.29 (0.06–10.29)	0.828		
Cardiovascular disease	6.39 (1.76–25.87)	**0.00535**	1.33 (0.32–6.00)	0.697
Cerebrovascular disease	7.96 (0.75–173.32)	0.093		
Carcinoma	12.18 (1.52–249.22)	**0.032**	2.57 (0.29–54.99)	0.434
Liver disease	4.02 (0.73–22.30)	0.0944		
Chronic obstructive lung disease	1.29 (0.19–5.81)	0.757		
**Laboratory findings**				
White blood cell count (×10^9^/L)	5.56 (2.69–12.43)	**9.59E-06**	7.11 (3.04–18.43)	**1.75E-05**
< 3.5	0.39 (0.09–1.16)	0.131	0.19 (0.03–0.75)	**0.034956**
3.5–9.5	1.00 (ref)		1.00 (ref)	
> 9.5	7.94 (3.19–21.18)	**1.43E-05**	8.38 (2.82–26.64)	**0.00018**
Lymphocyte count (×10^9^/L)	0.33 (0.16–0.64)	**0.00144**	0.49 (0.23–1.00)	0.0584
< 1.1	3.68 (1.83–7.94)	**0.000452**	2.41 (1.09–5.63)	**0.0342**
1.1–3.2	1.00 (ref)		1.00 (ref)	
> 3.2	4.18 (0.19–47.28)	0.258274	1.96 (0.08–26.78)	0.6238
Neutrophil count (x10^9^/L)	2.81 (1.54–5.31)	**0.00106**	3.00 (1.50–6.30)	**0.00259**
< 1.8	1.00 (0.35–2.49)	0.998	0.94 (0.28–2.77)	0.915091
1.8–6.3	1.00 (ref)		1.00 (ref)	
> 6.3	5.13 (2.33–11.44)	**5.11E-05**	6.03 (2.31–16.18)	**0.000268**
Monocyte count(x10^9^/L)	1.35 (0.63–2.81)	0.42501		
< 0.1	3.69 (0.14–94.79)	0.36		
0.1–0.6	1.00 (ref)			
> 0.6	1.53 (0.69–3.21)	0.274		
Platelet count (x10^9^/L)	1.16 (0.55–2.57)	0.698881		
< 125	1.18 (0.49–2.62)	0.693		
125–350	1.00 (ref)			
> 350	3.68 (0.66–20.52)	0.119		
NLR	3.92 (2.11–7.59)	**2.57E-05**	4.17 (2.00–9.26)	**0.000231**
< 0.78	NA^b^	0.986	NA	0.98938
0.78–3.53	1.00 (ref)		1.00 (ref)	
> 3.53	3.73 (1.96–7.32)	**8.52E-05**	3.78 (1.73–8.65)	**0.00113**
APTT(s)	1.90 (0.92–3.94)	0.082165		
< 21	NA	0.985		
21–37	1.00 (ref)			
> 37	1.48 (0.63–3.30)	0.352		
fibrinogen(g/L)	3.06 (1.58–5.99)	**0.000969**	2.69 (1.27–5.84)	**0.01063**
< 2	NA	0.9898	NA	0.9874
2–4	1.00 (ref)		1.00 (ref)	
> 4	2.90 (1.46–5.77)	**0.0023**	2.38 (1.07–5.33)	**0.0334**
D-dimer (μg/mL)	2.52 (1.29–4.96)	**0.00699**	2.52 (1.14–5.63)	**0.0228**
ESR (mm/1 h)	2.18 (1.05–4.54)	**0.0364**	2.54 (0.99–6.65)	0.052685
Procalcitonin (ng/mL)	4.40 (1.77–13.36)	**0.00337**	2.83 (1.03–9.34)	0.0598
CRP (mg/L)	2.02 (1.04–4.09)	**0.0434**	1.68 (0.76–3.90)	0.210687
Lactate dehydrogenase (U/L)	2.16 (1.16–4.13)	**0.0172**	1.47 (0.71–3.12)	0.30
< 91	0.34 (0.012–1.84)	0.3101	0.38 (0.02–2.56)	0.407692
91–230	1.00 (ref)		1.00 (ref)	
> 230	2.04 (0.98–4.22)	0.0539	1.29 (0.53–3.08)	0.563397
Creatine kinase (U/L)	1.77 (0.82–3.72)	0.136		
Creatinine (μmol/L)	1.67 (0.62–4.73)	0.3153		
< 44	1.27 (0.26–4.77)	0.7402		
44–112	1.00 (ref)			
> 112	2.95 (0.79–11.09)	0.0987		
BUN (mmol/L)	8.09 (2.84–29.37)	**0.000331**	9.20 (2.87–38.15)	**0.000611**
< 2.5	0.67 (0.10–2.76)	0.622078	0.54 (0.05–3.14)	0.54405
2.5–7.1	1.00 (ref)		1.00 (ref)	
> 7.1	18.16 (4.68–120.23)	**0.000229**	18.57 (4.23–133.27)	**0.00053**
AST (U/L)	2.30 (1.19–4.44)	**0.0131**	2.29 (1.05–5.03)	**0.036656**
ALT(U/L)	2.28 (1.09–4.70)	**0.0269**	3.67 (1.45–9.47)	**0.00615**
Total bilirubin (μmol/L)	5.71 (2.47–13.68)	**5.84E-05**	5.53 (2.12–14.92)	**0.000548**
< 3	NA	0.989	NA	0.990348
3–21	1.00 (ref)		1.00 (ref)	
> 21	5.60 (2.40–13.50)	**8.12E-05**	5.50 (2.10–14.88)	**0.000584**
SOFA score	2.88 (2.10–4.16)	**1.15E-09**	2.77 (1.95–4.16)	**1.20E-07**
0	1.00 (ref)		1.00 (ref)	
1	NA	0.99253	NA	0.992699
2	14.68 (3.43–102.01)	**0.00112**	10.72 (2.25–79.00)	**0.006283**
3	36.64 (8.66–256.66)	**1.34E-05**	35.09 (6.91–279.80)	**9.44E-05**
4	108.50 (16.22–1247.72)	**1.34E-05**	73.50 (9.45–9.52)	**0.000177**
5	403.00 (49.95–9761.98)	**2.01E-06**	474.72 (38.22–27,290.59)	**8.24E-05**
6	NA	0.99582	NA	0.996041
7	NA	0.99759	NA	0.998477

*OR* Odds ratio, *NLR* Neutrophil-to-lymphocyte ratio, *APTT* Activated partial thromboplastin time, *FIB* Fibrinogen, *ESR* Erythrocyte sedimentation rate, *PCT* Procalcitonin, *CRP* C-reactive protein, *LDH* Lactate dehydrogenase, *CK* Creatine kinase, *BUN* Blood urea nitrogen, *AST* Aspartate transaminase, *ALT* Alanine aminotransferase, *TBIL* Total bilirubin, *SOFA* Sequential Organ Failure Assessment

^a^ Independent risk factors were identified with multivariable logistic regression adjusted for potential confounders (details in supplementary material)

^b^Too few cases

Among all the symptoms on admission, dyspnea was highly associated with the critical illness, with an adjusted (for age, gender and comorbidities) OR (95% CI) of 46.01 (15.36–169.48), *p* < 0.0001 (Table 4). “Respiratory rate >24 breaths per min” also highly associated with critical illness, with an adjusted OR of 5.84 (1.53–23.01), *p* < 0.001 (Table 4).

Univariable analysis indicated association of critical illness with comorbidities hypertension, diabetes, cardiovascular disease and malignancy (Table 4). However, statistical significance was not achieved after adjustment for the influences of age and gender.

Abnormal counts of white blood cell was significantly correlated with critical illness. After adjusting for confounding factors, white blood cell counts > 9.5 X 10^9^/L was identified as a risk factor for critical illness, with an adjusted OR of 8.38 (2.82–26.64), *p* < 0.001. Neutrophilia was significantly correlated with critical illness. Neutrophil count > 6.3 X 10^9^/L was identified as a risk factor for critical illness with an adjusted OR of 6.03 (2.31–16.18), *p* < 0.001 (Table 4). Lymphopenia was associated with the critical illness, and lymphocyte < 1.1 X 10^9^/L was a risk factor with an adjusted OR of 2.41 (1.09–5.63), *p* < 0.05 (Table 4). Accordingly, elevated NLR was highly correlated with the critical illness, NLR > 3.53 was a risk factor for critical illness with an adjusted OR of 3.78 (1.73–8.65), *p* = 0.001. Other infection related markers, ESR, procalcitonin, CRP and LDH, was not significantly correlated with critical illness, after adjustment for age, gender and comorbidities.

Higher levels of the markers for coagulation function, fibrinogen and d-dimer, were correlated with the critical illness. Fibrinogen > 4 g/L and d-dimer > 0.55 μg/mL were risk factors for critical illness with adjusted ORs of 2.38 (1.07–5.33), *p* = 0.033, and 2.52 (1.14–5.63), *p* = 0.023, respectively (Table 4).

Higher levels of BUN, AST, ALT and total bilirubin were correlated with critical illness, with adjusted ORs of 9.20 (2.87–38.15), *p* < 0.001, 2.29 (1.05–5.03), *p* = 0.04; 3.67 (1.45–9.47), *p* < 0.01, 5.53 (2.12–14.92), *p* < 0.001, respectively (Table 4). Accordingly, higher SOFA score, an integrated reference for multi-organ failure, was highly correlated with critical illness, with an adjusted OR of 2.77 (1.95–4.16), *p* < 0.0001. SOFA scores 2 and greater was identified as risk factor for critical illness after adjustment for confounding factors.

**Fig. 2:**
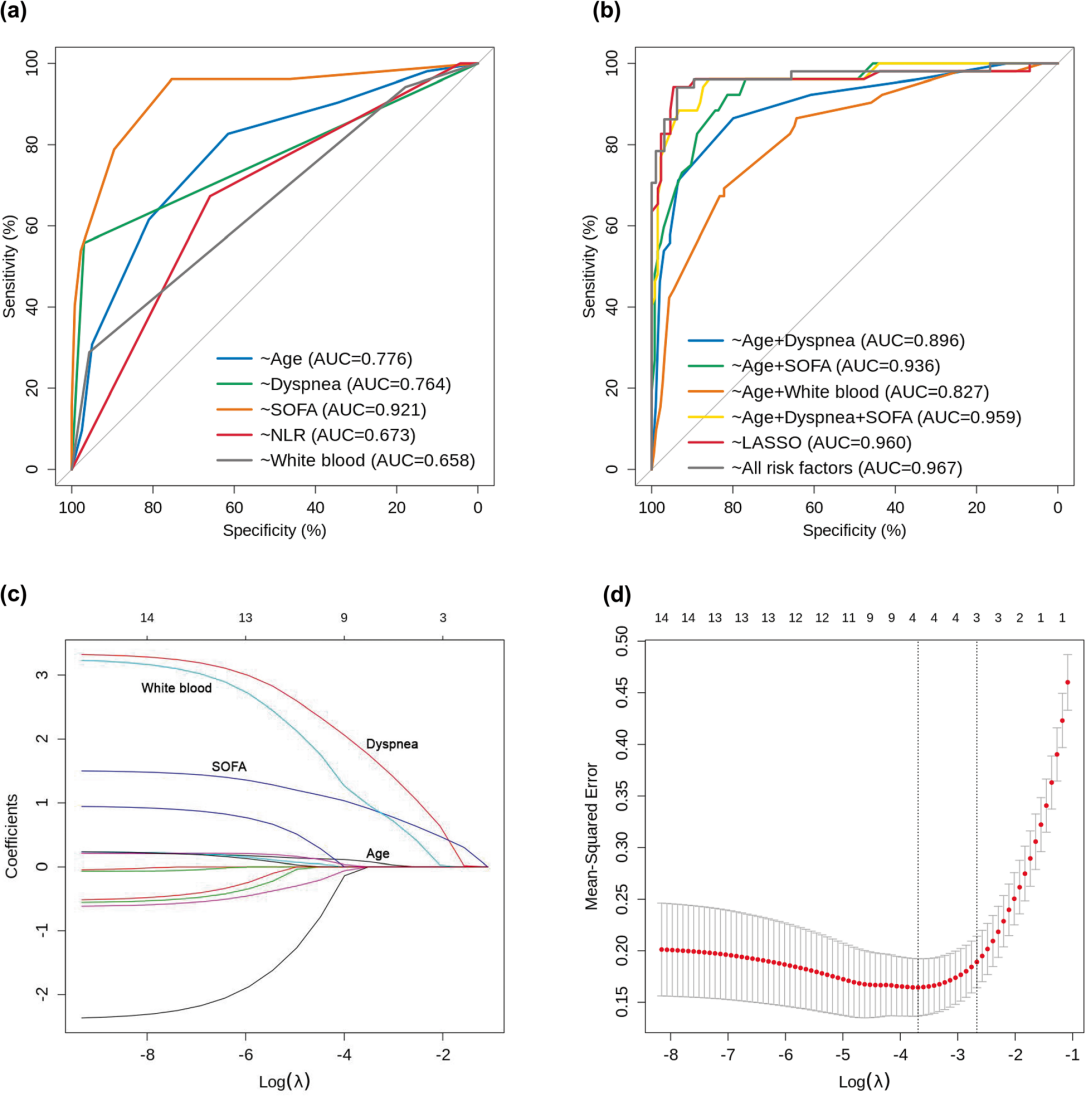
Risk factors for critical illness in COVID-19. Receiver-Operator Curve plots of individual risk factor models (**a**) and combined risk factor models (**b**) for predicting critical illness in SARS-CoV-2 infection. LASSO model was built based on age, dyspnea, SOFA score, and white blood cell count, risk factors selected by LASSO logistic regression. **c** LASSO coefficient profiles of the 14 risk factors of critical illness in SARS-CoV-2 infection. **d** Mean − Squared Error (MSE) plot of the LASSO model with different lambda. The best combination of risk factors was selected by LASSO logistic regression analyses, with four risk factors (labeled in (c)) selected by the lambda at which the minimal MSE was achieved

The Area Under the Receiver-Operator Curve (AUROC) was measured to evaluate the ability of the above critical illness associated risk factors for the prediction of adverse outcome (Supplementary Table S2). The individual factors with the highest AUROC values were SOFA score (0.921), age (0.776), dyspnea (0.764) and leukocytosis (0.658) (Fig. 2a). The AUROC for the combination of age and SOFA was 0.936 (Fig. 2b). The AUROC for the combination of all 14 risk factors in Table 4 was 0.967 (Fig. 2b).

Further, LASSO logistic regression analysis was conducted to select the best combination of predictors from 14 potential risk factors, and identified SOFA score, age, dyspnea, and white blood cell count and age as the most sensitive marker for the prediction of critical illness (Fig. 2c, d). The AUROC of the combined 4 factors was 0.960 (Fig. 2b, Supplementary Table S2).

## Discussion

Our retrospective multicenter study of 252 viral RNA positive COVID-19 patients identified SOFA score ≥ 2 best predicted critical illness on admission, followed by age older than 60, dyspnea, and several others that were significantly correlated with critical illness. Further, LASSO regression analysis identified the combination of SOFA score ≥ 2, age older than 60, dyspnea and white blood cell count greater than 9.5 X 10^9^/L as the best predictor for critical illness. Among these risk factors, SOFA score alone exhibited an AUROC of 0.921 for the prediction of critical illness. These risk factors may help with the early identification of the patients likely to require critical care services, which is valuable for optimal management of these patients and the clinical resources.

SOFA score is a sensitive diagnostic marker for sepsis and septic shock [11], and a good reference for multi-organ dysfunction [12]. In a recent retrospective multicenter study, SOFA was identified as a risk factor for mortality of adult COVID-19 patients, with an adjusted OR of 5.65 (2.61–12.23), *p* < 0.0001 [8]. In our study, although the OR for SOFA / critical illness is smaller than what was reported for SOFA / mortality, the SOFA score exhibited the best potential for the prediction of critical illness with an AUROC of 0.921.

SOFA is composed of PaO_2_/FiO_2_ representing the respiratory system, Glasgow score of consciousness representing the nervous system, mean blood pressure representing the cardiovascular system, total bilirubin representing hepatic system, platelet count representing coagulation system and creatinine representing renal system. Pioneering studies indicated that COVID-19 predominantly attacked respiratory system, leading to pneumonia related symptoms including cough, fever, elevated respiratory rate, dyspnea and ARDS, while symptoms related to other organ systems were rare [1, 2, 13]. In contrast, more recent studies reported frequent symptoms related to other organ systems such as diarrhea [14]. Relevant to SOFA score, in a study of critical illness, 40% of the critically ill patients had heart failure, and among these, many patients only had cardiovascular symptoms [15]. In addition, laboratory data from the studies of ours and the others [1, 2, 13] suggested tissue damage in renal, hepatic and coagulation systems. We observed that markers for the tissue damage in kidney and liver including BUN, AST, ALT, and total bilirubin were significantly correlated with critical illness after adjustment for confounding factors. Similarly, elevated markers for defective coagulation system, fibrinogen and d-dimer, were correlated with the critical status of COVID-19.

These observations echo the recent findings that SARS-CoV-2 virus was detected in multiple organs and tissues including bronchoalveolar lavage fluid, sputum, nasal and pharyngeal swabs, feces, blood [16] and the gastrointestinal tract [17]. The broad tropism of the viral infection are consistent with the universal tissue distribution of ACE2 [18, 19], the cellular receptor for SARS-CoV-2 [20]. However, further studies are needed to determine whether the multiple organ damage represented by the elevated SOFA score is due to direct tissue damage by viral infection or indirectly a consequence of hypoxia due to impaired respiratory system.

Previous study on COVID-19 identified older age, elevated d-dimer and higher SOFA score as the risk factors for mortality [8]. As expected, these mortality risk factors were found to be predictive for critical illness in our study. Importantly, additional 11 risk factors for critical illness were identified. Although SOFA score alone may have sufficient power to predict the disease outcome, the other risk factors such as dyspnea and leukocytosis are useful at times when components for the computation of SOFA score are missing.

Although many of the laboratory findings on admission were identified as predictive risk factors for critical illness, it is noteworthy that the majority of the laboratory findings on admission did not exceed the normal ranges. One reasonable explanation is that injuries caused by viral infection and inflammatory reactions were in the very early stages, so that some of the inflammation and injury related markers remained in the normal ranges. Limited by the retrospective nature of this study, it is not known whether these markers for inflammation and injuries increased around the time when severe symptoms presented. In addition, there seemed to be a mechanism for SARS-CoV-2 to escape the immune surveillance in certain patients as evidenced by the high prevalence of asymptomatic SARS-CoV-2 infected patients [21]. Therefore, another explanation is that inflammation and injuries were minimized because of immune evasion during the early disease course of critical COVID-19.

Previous studies reported that hypertension and diabetes were common in severe COVID-19 [3, 7]. However, statistical correlation analysis between comorbidities and critical illness was not performed in those studies. Our results showed that several comorbidities including hypertension and diabetes were more common in critical patients compared to non-critical patients. However, statistical significance was not achieved after adjusting for confounding factors. The impact of hypertension/diabetes on COVID-19 is complex. One interesting connection is that patients with hypertension and diabetes are usually treated with angiotensin-converting enzyme (ACE) inhibitors and angiotensin II type-I receptor blockers (ARBs), that induce the expression of ACE2 (the cellular receptor for SARS-CoV-2), thus likely to aggravate the disease course of COVID-19 [22]. On the other hand, the induction of ACE2 may be beneficial for COVID-19 patients exhibiting excessive inflammatory activities because ACE2 may reduce lung inflammation [23]. Therefore, accurate knowledge about the impact of hypertension/diabetes on COVID-19 awaits further study. Like other retrospective COVID-19 studies [3, 7], treatment data for comorbidities were not available in our study. And there are possibilities that medications for other comorbidities affect the disease course of COVID-19. For example, medications for inflammatory bowel diseases and autoimmune diseases may suppress patients’ immunity, causing a prolonged SARS-CoV-2 infection. This effect emphasizes the importance of adjusting the influences of the comorbidities in the multivariate regression for the identification of the risk factors of critical illness.

There are other limitations in our study. Firstly, being retrospective, our study was limited in significantly different sample sizes between the study groups. Fortunately, taking into account the imbalanced sample sizes of the two groups, statistical significance was achieved in correlation analysis between critical illness and many of the clinical features. In addition, we performed post-hoc power analyses and made sure that sufficient statistical power (> 0.9) was achieved in the establishment of SOFA, age, dyspnea and white blood cell count as the risk factors for critical illness. Secondly, the number of critically ill patients was relatively small. This prevented the stratification of the critical patients to identify the risk factors for mortality among the critical patients. Future studies with larger sample size should address this question. Thirdly, this retrospective study does not have data before patient admission. Some of the observed tissue damages might not be effects of SARS-CoV-2 infection. They could be the consequences of comorbidities. However, our data indicated that the overall frequency of comorbidities were small (Table 1). Therefore, SARS-COV-2 infection likely played the major role in tissue damage. More importantly, the influence of comorbidities were adjusted in the multivariate regression analyses for the identification of the risk factors for critical illness. This study is also limited in collecting laboratory data only on admission and before discharge. Since the majority of the laboratory data on admission were in the normal ranges and the data before discharge also a confirmation of patients recovering from COVID-19, it was not a surprise that no significant change on laboratory data was observed.

## Conclusions

We identified demographic features, clinical symptoms and laboratory measurements on admission that were correlated with critical illness in COVID-19. Further, SOFA score ≥ 2, age > 60, dyspnea and white blood cell count > 9.5 X 10^9^/L were identified as the best combination of risk factors. Our findings advocate the use of these risk factors for the prediction of critical illness in the management of the patients and health care resources.

## Supplementary information

**Additional file 1: Supplementary Table S1.** Reference value of variables and their potential confounders adjusted in multivariable logistic regression.

**Additional file 2: Supplementary Table S2.** Performance of different models for predicting critical illness in SARS-CoV-2 infection.

**Additional file 3: Supplementary Table S3.** Laboratory findings of all critically ill patients on admission and before discharge.

**Additional file 4: Supplementary Table S4.** Laboratory findings of critically ill patients on admission, different hospitals.

**Additional file 5: Supplementary Table S5.** Imaging features on admission, different hospitals.

## Data Availability

The data that support the findings of this study are available from the corresponding author upon reasonable request.

## Abbreviations

ALT: Alanine aminotransferase
AST: Aspartate transaminase
APTT: Activated partial thromboplastin time
AUROC: The Area under the Receiver Operating Characteristic
BUN: Blood urea nitrogen
ARDS: Acute respiratory distress syndrome
CK: Creatine kinase
COVID-19: Coronavirus Disease 2019
CRP: C-reactive protein
CT: Computed tomography
ECMO: Extracorporeal membrane oxygenation
ESR: Erythrocyte sedimentation rate
FiO_2_: Fraction of inspired oxygen
FIB: Fibrinogen
ICU: Intensive care unit
LDH: Lactate dehydrogenase
NLR: Neutrophil-to-lymphocyte ratio
SARS-CoV-2: Severe acute respiratory syndrome-related coronavirus-2
SOFA: Sequential Organ Failure Assessment
