# Supplementary material for: Risk factors for the critical illness in SARS-CoV-2 infection: a multicenter retrospective cohort study
Source: Respir Res. 2020 Oct 21;21:277. doi: 10.1186/s12931-020-01492-z (PMC7576549; doi:10.1186/s12931-020-01492-z)
Supplement: Supplementary file 1 — Additional file 1: Supplementary Table S1. Reference value of variables and their potential confounders adjusted in multivariable logistic regression. [file 12931_2020_1492_MOESM1_ESM.pdf]

Supplementary Table S1. Reference value of variables and their potential confounders adjusted in multivariable logistic regression

	Reference value	Confounders adjusted in multivariable logistic regression
**Demographics and clinical characteristics**		
Age，years	<30; 30-39; 39-49; 49-59; 39-69; 69-79; >79	-
Female sex (vs male)	-	-
Dyspnea (vs not dyspnea)	-	Age, Sex, all comorbidities
Respiratory rate >24 breaths per min (vs respiratory rate ≤24 breaths per min)	-	Age, Sex, all comorbidities, Dyspnea
**Comorbidity present (vs not present)**		
Hypertension	-	Age, Sex
Diabetes	-	Age, Sex
Digestive tract disease	-	Age, Sex
Cardiovascular disease	-	Age, Sex
Cerebrovascular disease	-	Age, Sex
Carcinoma	-	Age, Sex
Liver disease	-	Age, Sex
Chronic obstructive lung disease	-	Age, Sex
SOFA score	-	Age, Sex
**Laboratory findings**		
White blood cell count ($X10^9$/L)	3.5-9.5	Age, Sex, all comorbidities
Lymphocyte count ($X10^9$/L)	1.1-3.2	Age, Sex, all comorbidities
Neutrophil count ($x10^9$/L)	1.8-6.3	Age, Sex, all comorbidities
Monocyte count ($x10^9$/L)	0.1-0.6	Age, Sex, all comorbidities
Platelet count ($x10^9$/L)	125-350	Age, Sex, all comorbidities
APTT (s)	21-37	Age, Sex, all comorbidities
FIB (g/L)	2-4	Age, Sex, all comorbidities
D-dimer (μg/mL)	0-0.55	Age, Sex, all comorbidities
ESR (mm/1h)	0-30	Age, Sex, all comorbidities
PCT (ng/mL)	0-0.05	Age, Sex, all comorbidities
CRP (mg/L)	0-10	Age, Sex, all comorbidities
NLR	0.78-3.53	Age, Sex, all comorbidities
LDH (U/L)	91-230	Age, Sex, all comorbidities
CK (U/L)	<171	Age, Sex, all comorbidities
Creatinine (μmol/L)	44-112	Age, Sex, all comorbidities
BUN(mmol/L)	2.5-7.1	Age, Sex, all comorbidities
AST (U/L)	0-40	Age, Sex, all comorbidities

ALT (U/L)	0-50	Age, Sex, all comorbidities
TBIL (μmol/L)	3-21	Age, Sex, all comorbidities

NLR=neutrophil-to-lymphocyte ratio. FIB=fibrinogen. ESR=Erythrocyte sedimentation rate. PCT=Procalcitonin. CRP=C-reactive protein. LDH=Lactate dehydrogenase. CK=Creatine kinase. BUN=blood urea nitrogen. AST=aspartate transaminase. ALT=alanine aminotransferase. TBIL=Total bilirubin.
