# Supplementary material for: Risk factors for the critical illness in SARS-CoV-2 infection: a multicenter retrospective cohort study
Source: Respir Res. 2020 Oct 21;21:277. doi: 10.1186/s12931-020-01492-z (PMC7576549; doi:10.1186/s12931-020-01492-z)
Supplement: Supplementary file 2 — Additional file 2: Supplementary Table S2. Performance of different models for predicting critical illness in SARS-CoV-2 infection. [file 12931_2020_1492_MOESM2_ESM.pdf]

Supplementary Table S2. Performance of different models for predicting critical illness in SARS-CoV-2 infection

Risk factor models for critical illness	AUC (95%CI)
**Single risk factor models**	
Age，years	0.776 (0.705-0.848)
Dyspnea (vs not dyspnea)	0.764 (0.695-0.833)
Respiratory rate >24 breaths per min (vs respiratory rate ≤24 breaths per min)	0.665 (0.598-0.732)
SOFA score	0.921 (0.875-0.957)
White blood cell count ($X10^9$/L)	0.658 (0.590-0.727)
Lymphocyte count ($X10^9$/L)	0.635 (0.565-0.705)
Neutrophil count ($x10^9$/L)	0.620 (0.540-0.700)
FIB (g/L)	0.625 (0.550-0.700)
D-dimer (μg/mL)	0.602 (0.525-0.679)
NLR	0.673 (0.602-0.744)
BUN (mmol/L)	0.618 (0.552-0.685)
AST (U/L)	0.593 (0.516-0.669)
ALT (U/L)	0.572 (0.502-0.642)
TBIL (μmol/L)	0.615 (0.551-0.680)
**Combination models**	
Age + Dyspnea	0.896 (0.844-9.948)
Age + SOFA score	0.936 (0.900-0.973)
Age + NLR	0.816 (0.751-0.881)
Age + White blood	0.827 (0.763-0.891)
Age + Dyspnea + SOFA score	0.959 (0.927-0.991)
Age + Dyspnea + NLR	0.904 (0.858-0.950)
Age + SOFA score + NLR	0.835 (0.897-0.973)
Age + Dyspnea + SOFA score + NLR	0.957 (0.922-0.991)
*Age + Dyspnea + SOFA score + White blood	0.960 (0.919-1.000)
#ALL	0.967 (0.931-1.000)

FIB=fibrinogen. NLR=neutrophil-to-lymphocyte ratio. BUN=blood urea nitrogen. AST=aspartate transaminase. ALT=alanine aminotransferase. TBIL=Total bilirubin.

Models were constructed using multivariable logistic regression.

* This model used 4 important risk factors selected by LASSO logistic regression analysis.

# This model used all 14 risk factors.
