# Supplementary material for: Risk factors for the critical illness in SARS-CoV-2 infection: a multicenter retrospective cohort study
Source: Respir Res. 2020 Oct 21;21:277. doi: 10.1186/s12931-020-01492-z (PMC7576549; doi:10.1186/s12931-020-01492-z)
Supplement: Supplementary file 3 — Additional file 3: Supplementary Table S3. Laboratory findings of all critically ill patients on admission and before discharge. [file 12931_2020_1492_MOESM3_ESM.pdf]

Supplementary table S3. Laboratory findings of all critically ill patients on admission and before discharge

	Reference values	On admission	Before discharge	p value
**Laboratory findings**				
White blood cell count (X 10^9/L)	3.50-9.50	6.45 (4.53-10.91)	7.89 (4.65-11.49)	0.764
Lymphocyte count (X 10^9/L)	1.10-3.20	0.79 (0.58-1.07)	1.00 (0.59-1.32)	0.117
Neutrophil count (X 10^9/L)	1.80-6.30	4.87 (2.86-8.37)	4.91 (3.22-8.04)	0.557
Monocyte count (X 10^9/L)	0.10-0.60	0.36 (0.24-0.54)	0.41 (0.28-0.58)	0.633
Platelet count (X 10^9/L)	125.00-350.00	194.00 (148.00-274.50)	212.50 (157.00-296.75)	0.150
NLR	0.78-3.53	6.51 (2.54-14.12)	5.07 (2.27-10.97)	0.310
APTT (s)	21.00-37.00	34.20 (28.61-36.39)	34.40 (25.92-38.08)	0.767
FIB (g/L)	2.00-4.00	3.32 (2.68-4.10)	3.68 (2.64-4.56)	0.122
D-dimer (μg/mL)	0.00-0.55	0.40 (0.28-0.61)	0.56 (0.35-1.48)	0.577
ESR (mm/1h)	0.00-30.00	31.00 (23.75-45.25)	43.00 (26.00-52.00)	0.083
PCT (ng/mL)	0.00-0.50	0.24 (0.10-0.37)	0.23 (0.10-0.37)	0.930
CRP(mg/L)	0.00-10.00	24.70 (6.89-100.19)	28.67 (6.29-92.31)	0.350
LDH (U/L)	91.00-230.00	226.00 (183.00-323.10)	214.00 (168.50-321.50)	0.892
CK (U/L)	25.00-200.00	150.00 (47.85-186.50)	65.6 (29.80-181.00)	0.248
Creatinine (μmol/L)	44.00-112.00	74.19 (62.05-86.00)	69.70 (57.83-91.25)	0.397
BUN (mmol/L)	2.50-7.10	5.41 (4.16-6.90)	6.41 (4.70-7.88)	0.243
AST (U/L)	0.00-40.00	37.00 (25.70-56.00)	45.00 (29.18-64.75)	**0.038**
ALT (U/L)	0.00-50.00	36.45 (22.48-56.03)	46.00 (33.78-80.25)	**0.008**
TBIL (μmol/L)	3.00-21.00	17.20 (8.65-23.53)	19.40 (12.90-23.50)	0.243

Data are median (IQR). p values are from Wilcoxon test. NLR=neutrophil-to-lymphocyte ratio. APTT=activated partial thromboplastin time. FIB=fibrinogen. ESR=Erythrocyte sedimentation rate. PCT=Procalcitonin. CRP=C-reactive protein. LDH=Lactate dehydrogenase. CK=Creatine kinase. BUN= blood urea nitrogen. AST=aspartate transaminase. ALT=alanine aminotransferase. TBIL=Total bilirubin.
