# Supplementary material for: Risk factors for the critical illness in SARS-CoV-2 infection: a multicenter retrospective cohort study
Source: Respir Res. 2020 Oct 21;21:277. doi: 10.1186/s12931-020-01492-z (PMC7576549; doi:10.1186/s12931-020-01492-z)
Supplement: Supplementary file 4 — Additional file 4: Supplementary Table S4. Laboratory findings of critically ill patients on admission, different hospitals. [file 12931_2020_1492_MOESM4_ESM.pdf]

Supplementary Table S4, Laboratory findings of critically ill patients on admission, different hospitals

	Reference values	All critically ill (n=52)	Critically ill, Dongguan People's Hospital (n=13)	Critically ill, Jingzhou Hospital of Traditional Chinese Medicine (n=23)	Critically ill, Jingzhou Central Hospital (n=15)	p value
**Laboratory findings**						
White blood cell count (X 10^9/L)	3.50-9.50	6.45 (4.53-10.91)	5.69 (4.84-7.17)	5.46 (3.80-7.53)	11.40 (8.82-13.69)	**0.002**
Lymphocyte count (X 10^9/L)	1.10-3.20	0.79 (0.58-1.07)	0.75 (0.40-1.09)	0.78 (0.57-1.07)	0.83 (0.65-1.10)	0.771
Neutrophil count (X 10^9/L)	1.80-6.30	4.87 (2.86-8.37)	4.37 (3.33-6.43)	3.55 (1.53-5.74)	8.42 (5.91-12.80)	**0.002**
Monocyte count (X 10^9/L)	0.10-0.60	0.36 (0.24-0.54)	0.36 (0.32-0.42)	0.35 (0.17-0.54)	0.34 (0.24-0.82)	0.591
Platelet count (X 10^9/L)	125.00-350.00	194.00 (148.00-274.50)	162.00 (148.00-251.00)	178.00 (124.00-254.00)	231.00 (201.00-323.00)	**0.024**
NLR	0.78-3.53	6.51 (2.54-14.12)	6.43 (2.74-14.54)	4.30 (1.90-9.71)	11.71 (4.65-17.54)	0.066
APTT (s)	21.00-37.00	34.20 (28.61-36.39)	35.70 (34.20-40.55)	30.41 (24.65-35.70)	33.54 (30.20-36.40)	0.062
FIB (g/L)	2.00-4.00	3.70 (3.11-4.86)	4.62 (3.33-5.51)	3.57 (3.25-4.53)	3.76 (2.67-5.11)	0.287
D-dimer (μg/mL)	0.00-0.55	0.50 (0.31-1.30)	1.17 (0.43-1.92)	0.38 (0.28-1.16)	0.54 (0.35-1.02)	0.201
ESR (mm/1h)	0.00-30.00	31.00 (23.75-45.25)	15.00 (5.50-58.75)	30.00 (25.00-65.00)	37.00 (24.00-44.00)	0.236
PCT (ng/mL)	0.00-0.50	0.24 (0.10-0.37)	0.11 (0.10-0.17)	0.34 (0.13-0.40)	0.30 (0.23-0.42)	**0.017**
CRP(mg/L)	0.00-10.00	24.70 (6.89-100.19)	17.86 (5.00-65.02)	34.06 (11.21-113.63）	39.67 (6.89-130.00)	0.268
LDH (U/L)	91.00-230.00	226.00 (183.00-323.10)	202.55 (178.63-288.96)	226.00 (184.50-483.50)	250.50 (165.00-342.00)	0.584
CK (U/L)	25.00-200.00	150.00 (47.85-186.50)	47.85 (35.70-70.08)	175.50 (152.00-380.25)	108.75 (26.43-350.75)	**0.001**
Creatinine (μmol/L)	44.00-112.00	74.19 (62.05-86.00)	64.30 (46.40-71.60)	84.60 (68.90-94.55)	63.20 (54.30-79.00)	**0.001**
BUN (mmol/L)	2.50-7.10	5.41 (4.16-6.90)	4.90 (3.80-6.00)	6.01 (5.01-8.98)	5.32 (4.16-6.71)	0.089
AST (U/L)	0.00-40.00	37.00 (25.70-56.00)	24.70 (18.70-29.20)	41.00 (31.00-65.00)	45.00 (35.00-76.00）	**0.001**
ALT (U/L)	0.00-50.00	36.45 (22.48-56.03)	22.40 (17.65-53.65)	37.00 (26.00-49.00)	51.00 (32.10-89.00)	0.057

TBIL (µmol/L)	3.00-21.00	17.20 (8.65-23.53)	18.10 (9.25-21.10)	15.30 (7.80-17.80)	28.90 (20.10-32.80)	**0.001**

Data are median (IQR). p values are from Kruskal-Wallis test. NLR=neutrophil-to-lymphocyte ratio. APTT=activated partial thromboplastin time. FIB=fibrinogen. ESR=Erythrocyte sedimentation rate. PCT=Procalcitonin. CRP=C-reactive protein. LDH=Lactate dehydrogenase. CK=Creatine kinase. BUN= blood urea nitrogen. AST=aspartate transaminase. ALT=alanine aminotransferase. TBIL=Total bilirubin.
