# Supplementary material for: Risk factors for the critical illness in SARS-CoV-2 infection: a multicenter retrospective cohort study
Source: Respir Res. 2020 Oct 21;21:277. doi: 10.1186/s12931-020-01492-z (PMC7576549; doi:10.1186/s12931-020-01492-z)
Supplement: Supplementary file 5 — Additional file 5: Supplementary Table S5. Imaging features on admission, different hospitals. [file 12931_2020_1492_MOESM5_ESM.pdf]

Supplementary Table S5, Imaging features on admission, different hospitals

	Jingzhou Hospital of Traditional Chinese Medicine (n=103)			Jingzhou Central Hospital (n=21)			Jieyang People's Hospital (n=8)		
	Non-critically ill (n=80)	Critically ill (n=23)	p value	Non-critically ill (n=6)	Critically ill (n=15)	p value	Non-critically ill (n=7)	Critically ill (n=1)	p value
**Imaging features**									
Ground-glass opacity	11 (13.8)	7 (30.4)	0.063	1 (16.7)	2 (13.3)	0.844	1 (14.3)	0 (0)	0.686
Unilateral pulmonary abnormality	16 (20.0)	1 (4.3)	0.075	1 (16.7)	0 (0)	0.105	3 (42.9)	0 (0)	0.408
Bilateral pulmonary abnormality	60 (75.0)	21 (91.3)	0.093	1 (16.7)	4 (26.7)	0.627	4 (57.1)	1 (100.0)	0.408

Data are n (%). p values are from χ^2 test.
